# Insights into Target Gas–Oxygen Interactions in Highly Sensitive Gas Sensors Using Data-Driven Methods

**DOI:** 10.1021/acssensors.4c01284

**Published:** 2024-11-12

**Authors:** Kyusung Kim, Phuwadej Pornaroontham, Hojung Yun, Sungmin Kim, Pilgyu Choi, Yoshitake Masuda

**Affiliations:** 1 Institute of Material Innovation, Institutes of Innovation for Future Society, Nagoya University, Nagoya 464-8601, Japan; 2 Department of Chemical Technology, Faculty of Science, Chulalongkorn University, Bangkok 10330, Thailand; 3 Machine learning for Polymers and Materials Discovery Research Unit, Chulalongkorn University, Bangkok 10330, Thailand; 4 Department of Sustainable Materials and Technology for Industries, Faculty of Engineering, Nagoya University, Nagoya 464-8601, Japan; 5 Korea Institute of Industrial Technology, Surface R&D Group, 156, Gaetbeol-ro, Yeonsu-gu, Incheon 21999, Republic of Korea; 6 National Institute of Advanced Industrial Science and Technology (AIST), 2266-98 Anagahora, Shimoshidami, Moriyama ,Nagoya463-8560, Japan

**Keywords:** SnO_2_, nanosheets, gas sensors, flow rate, clustering

## Abstract

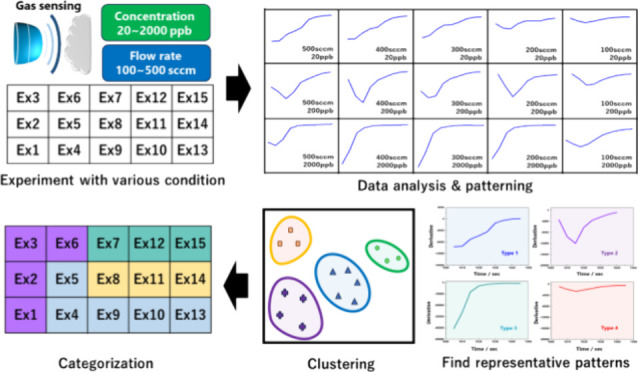

In the gas-sensing mechanism of a metal-oxide-semiconductor (n-type) gas sensor, oxygen adsorption or desorption on the oxide surface leads to an increase or decrease in the resistance of the gas sensor system. Additionally, oxygen can be adsorbed again at the location where initially adsorbed oxygen reacted with the target gas. Thus, the adsorption–desorption equilibrium of the reducing gas on the oxide surface is a significant factor in determining the sensitivity and reaction rate. In particular, for ultralow-concentration gas measurements, the relative concentration of oxygen was very high. To design an ultrasensitive gas sensor, not only the reaction of the target gas but also the competing reaction between the target gas and oxygen must be considered. Although qualitative investigations of these complex relationships have been performed according to the gas concentration and flow rate, reliable quantitative results are limited. In this study, a quantitative approach was used to understand the correlation between oxygen and a target gas by applying data analysis methods. We investigated the behavior of oxygen and the target molecules depending on the gas concentration and flow rate using the parts per billion level of the acetone gas sensor. Initial response data according to various detection conditions were processed using principal component analysis and K-means clustering; as a result, four types of reaction behaviors were inferred for 15 types of reaction conditions. Furthermore, the response time, depending on the detection conditions, can be distinguished using the suggested categorization. Our investigation suggests a possibility beyond simple optimization through the data analysis of the gas-sensing results.

Metal-oxide semiconductor gas sensors use resistance changes owing to the free electrons generated when the target molecules react on their surfaces.^1,2^ During this process, the equilibrium between adsorption and desorption is important because other surrounding molecules can react again with the oxide to increase resistance. Many researchers have studied the effects of the operating temperature and gas flow rate on gas sensors.^3−7^ In particular, optimization of the temperature has been extensively reported because it has a significant influence on the electronic conductivity of the sensing material via thermal excitation and surface adsorption/desorption behavior;^8^ however, the impact of flow rate variation has not been investigated. The operating temperature is closely related to the conductivity of the sensing material and thermal decomposition of the target molecule, whereas the effect of the flow rate is considered to be a simple variance. However, the gas response can be determined not only by the reaction of the target molecules but also by their competition with the oxygen molecules. Hence, the effect of the flow rate, which determines the absolute number of reaction molecules, is important for ultrasensitive sensors. Therefore, the gas concentration and flow rate can significantly affect this balance. In general, a qualitative understanding of gas sensing was obtained by changing these parameters, but detailed molecular behavior and quantitative information were not revealed.^6^

Recently, various data processing and analysis methods have been used to solve numerous problems.^9−11^ In this study, data science was applied to quantitatively investigate the effects of the gas concentration and flow rate variations on molecular reactions at the surface. As the sensor material, SnO_2_ was chosen, which is a typical n-type wide bandgap semiconductor with high conductivity and good stability. Moreover, for detecting low concentration gas, the SnO_2_ nanosheet structure was employed, utilizing the highly reactive (101) crystal plane as the main reaction site.^12^ In particular, the focus was on the change in resistance during the initial step. In general, the resistance signal requires some time to stabilize after a rapid change in the initial step as the injected target gas reacts with the sensor. This stabilization time is determined by the reaction between the target molecule and oxygen molecule. Through differential values from the initial dynamic curve of gas sensing, specific patterns of curves were confirmed, depending on the concentration and flow rate of the gas. To quantify differences in patterns, principal component analysis (PCA) and K-mean clustering were applied. Based on the correlation between these classified patterns and variables, we proposed a change in gas adsorption and desorption at the surface according to the gas concentration and flow rate.

## Experimental Procedure

### Synthesis of SnO_2_ Nanosheets

**Figure 1:**
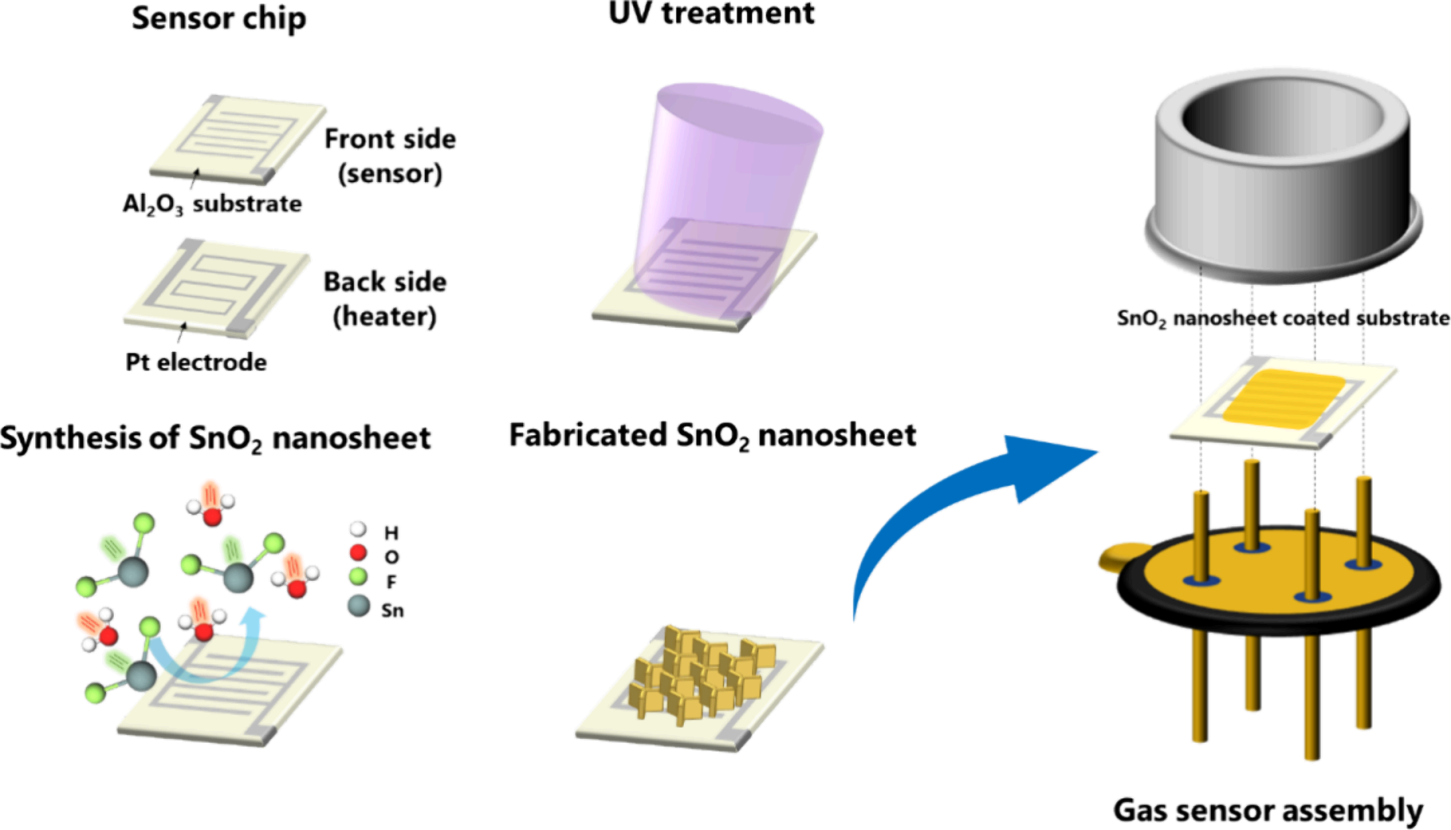
Schematic of the Pt-interdigitated electrode chip and the synthesis procedure of SnO_2_ nanosheets on the chip and assembly of the gas sensor.

SnO_2_ nanosheets were synthesized on a Pt-interdigitated electrode chip as the substrate, as shown in Figure 1. First, a vacuum ultraviolet (VUV) treatment of the surface of the sensor chip was performed to promote the initial nucleation and growth of the SnO_2_ nanosheets. Simultaneously, a SnF_2_ solution was prepared by mixing 870.6 mg of SnF_2_ with 200 mL of distilled water. The prepared sensor chip was placed in the SnF_2_ solution in a polypropylene vessel and kept at 90 °C in an oven for 6 h. After the formation of SnO_2_ nanosheets on the chip, the surface of the sample was rinsed with distilled water.

### Characterizations

The morphology of the SnO_2_ nanosheets was observed using field-emission scanning electron microscopy (FE-SEM; JSM-6335FM, JEOL, Japan), and its detailed structure was revealed using transmission electron microscopy (TEM; Tecnai Osiris, FEI, USA) and high-resolution transmission electron microscopy (HR-TEM; Tecnai Osiris, FEI, USA). The crystal structure was confirmed by X-ray diffraction (XRD, SmartLab, Rigaku Co. Ltd., Japan) with Cu Kα (λ = 1.5418 Å) radiation at 40 kV and 30 mA. Diffraction patterns were acquired in the range of 20–80°. The chemical binding energies of the surfaces were investigated by using X-ray photoelectron spectroscopy (XPS; ESCA, Shimadzu, Japan).

### Gas-Sensing Measurements

Gas-sensing measurements were performed by using a gas mixer (MU-3609, Horiba, Japan) and a resistance meter. The temperature of the gas sensor was set at 280 °C. In addition, the gas was mixed using pure N_2_ (99.99995%) and N_2_ + acetone (0.1 ppm) gas bombe without any humidity control. The size of the chamber was 30 (L) × 30 (W) × 24 (H) mm. The flow rate and acetone concentration were controlled in ranges of 100–500 sccm and 20, 200, and 2000 ppb, respectively. To stabilize the initial resistance of the sensor, it was pretreated for 2 h for all measurements. The gas response is defined as *R*_a_/*R*_g_, where *R*_a_ and *R*_g_ are the electrical resistances in air and the target gas, respectively.

### Cluster Analysis

To group the data based on similarity, they were reduced to two dimensions using PCA. This was performed using the sklearn.decomposition.PCA package from scikit-learn version 1.2.2,^13^ a well-known library for machine learning in Python programming (Python version 3.10). Principal component scores (%PC) and eigenvalues were obtained using the pca.explained.variance_ratio_ and pca.explained.variance_ attributes. The covariance matrix can be obtained by using the get.covariance() method. The first two principal components were selected and passed to an unsupervised machine-learning algorithm for K-means clustering. The algorithm first randomly selects initial *k* centroids, where *k* is a predefined number of clusters and the centroid represents the center points of each cluster. It then iteratively assigns each data point to the nearest centroid based on the Euclidean distance between the data point and the centroid and recalculates the centroid based on the mean of all the data points assigned to it. This process was repeated until the centroids no longer moved significantly, indicating convergence. This was performed using the sklearn.cluster.K-means package. The number of predefined clusters was set from two to six, and the optimal number of clusters was evaluated by the elbow method using two matrices: within-cluster sum of squared errors (WSS) and Calinski–Harabasz index (CH).^14^ For a given data set *X* with *n* data points and a desired number of clusters *k*, the K-means clustering algorithm aims to find *k* cluster centroids *C* = *c*_1_, *c*_2_,..., *c*_*k*_ that minimize the total within-cluster sum of squares, defined as
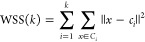
where *C*_*i*_ is the set of data points assigned to the *i*th centroid *c*_*i*_ and || *x* – *c*_*i*_ || is the Euclidean distance between data point *x* and centroid *c*_*i*_. The CH index is a measure of cluster validity that evaluates the quality of a clustering solution based on the ratio of the between-cluster sum of squares (BSS) to the within-cluster sum of squares. A higher CH index indicates a better clustering solution. The CH index was calculated using the following formula:
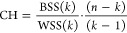

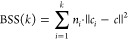
where *k* is the number of clusters, *n* is the total number of data points, *n*_*i*_ is the number of data points in the *i*th cluster, *c*_*i*_ is the centroid of the *i*th cluster, *c* is the overall centroid, and || *c*_*i*_ – *c* ||^2^ is the squared Euclidean distance. The visualization of the elbow method can be implemented using the Yellowbrick library^15^ through the KElbowVisualizer module.

## Results and Discussion

### Characterizations of the SnO_2_ Nanosheet

**Figure 2:**
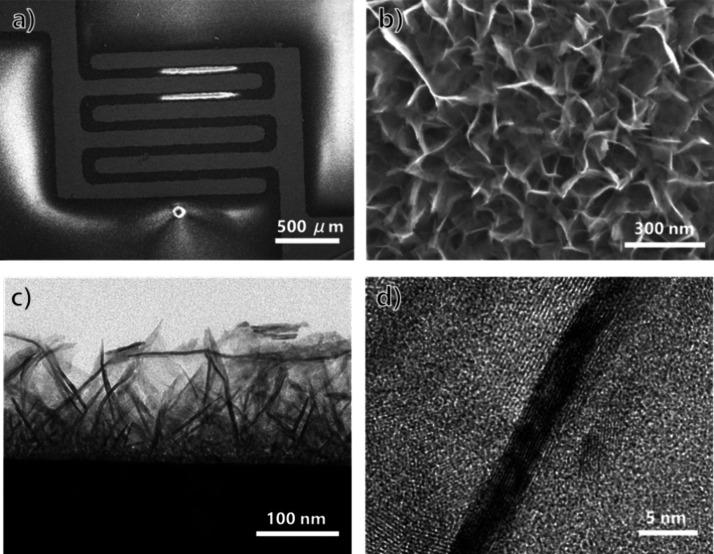
(a) Top view of the sensor chip. (b) Top-view FE-SEM image and (c) the cross-sectional TEM image of SnO_2_ nanosheets. (d) HR-TEM image of a single SnO_2_ nanosheet.

The morphologies of the SnO_2_ nanosheets were observed by using SEM and TEM. Figure 2a shows the top view of the sensor chip covered by the SnO_2_ nanosheets, and Figure 2b presents the enlarged nanostructures on the surface. A vertically structured nanosheet can be seen, and the edges of adjacent sheets are interconnected. The uniformly dispersed nanosheet structure without agglomeration is due to these interconnections. The TEM image in Figure 2c shows that the thin nanosheets grew vertically with a thickness of approximately 150 nm. The single SnO_2_ nanosheet visualized via HR-TEM (Figure 2d) exhibits a lattice distance of *d* = 0.264 nm, corresponding to the SnO_2_ (101) planes.

**Figure 3:**
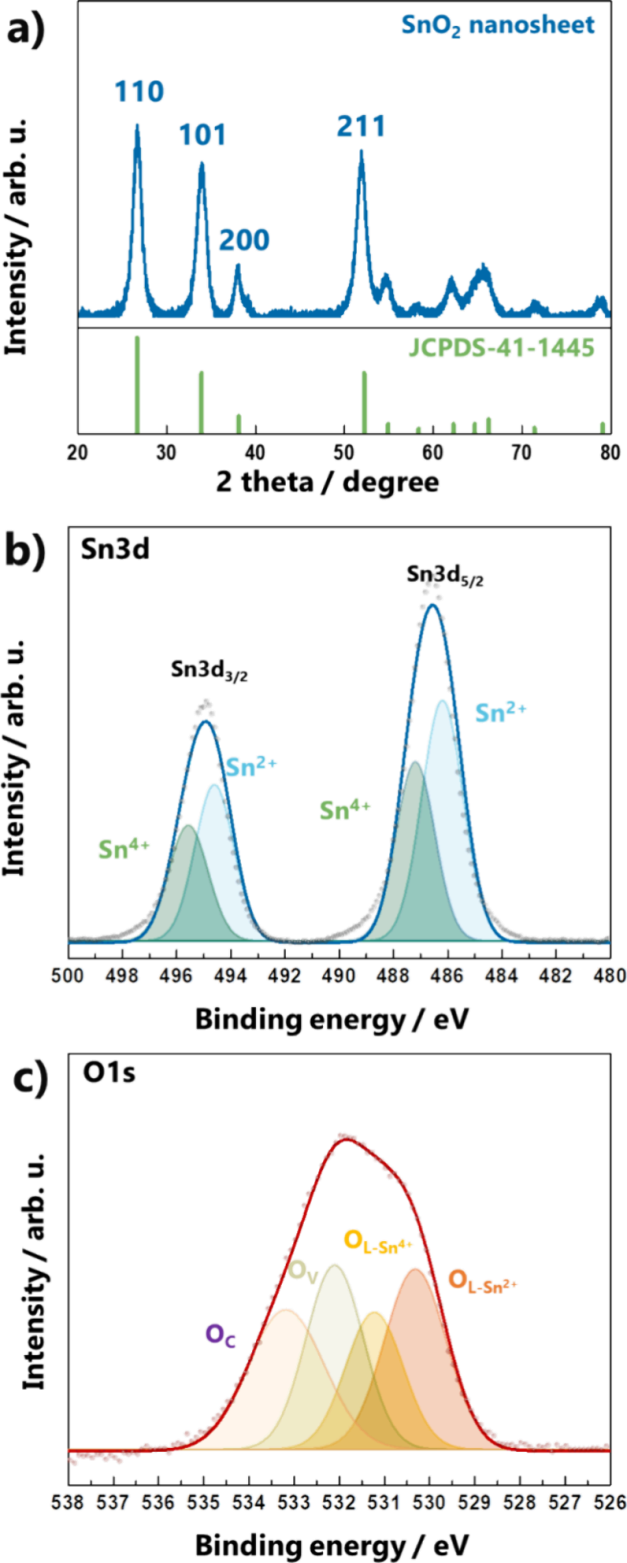
(a) XRD patterns and high-resolution XPS spectra of (b) Sn 3d and (c) O 1s of SnO_2_ nanosheets.

The crystalline structure of the SnO_2_ nanosheets was revealed by XRD, as shown in Figure 3a. The diffraction pattern corresponds to the tetragonal cassiterite structure of SnO_2_ (JCPDS no. 41-1445). The atomic compositions and chemical states of the surfaces were analyzed by XPS. The high-resolution Sn 3d and O 1s spectra are presented in Figure 3b and Figure 3c, respectively. Sn 3d peaks were observed at 486.2 eV (Sn 3d_5/2_) and 494.6 eV (Sn 3d_3/2_), and both peaks were deconvoluted into Sn^2+^ and Sn^4+^. The (101) facet allows the coexistence of Sn^2+^ and Sn^4+^ states, and the Sn^4+^ state is more stable than the Sn^2+^ state owing to the inert pair effect.^16^ Therefore, the Sn^2+^ state in the (101) facet could contribute to the superior gas detection performance. The asymmetric O 1s spectrum contains four peaks with binding energies of 530.3, 531.2, 532.1, and 533.2 eV. These peaks are attributed to oxygen in the lattice (for Sn^2+^ and Sn^4+^), vacancies, and chemisorbed species, respectively.^15−17^ In particular, vacancies and chemisorbed species are important for gas detection.^17−19^ In previous studies, SnO_2_ nanosheets exhibited excellent performance for acetone gas sensing.^12,20,21^ This was attributed to the enhanced generation of the chemisorbed oxygen species. Our SnO_2_ nanosheet is suitable for the chemisorption of oxygen atoms because it mainly exposes the (101) facet containing the Sn^2+^ state. This state can lead to the formation of chemisorbed oxygen species owing to their high reactivity. Consequently, a large proportion of the O_C_ peak was observed in the XPS spectra.

### Acetone Gas Detection of the SnO_2_ Nanosheet

**Figure 4:**
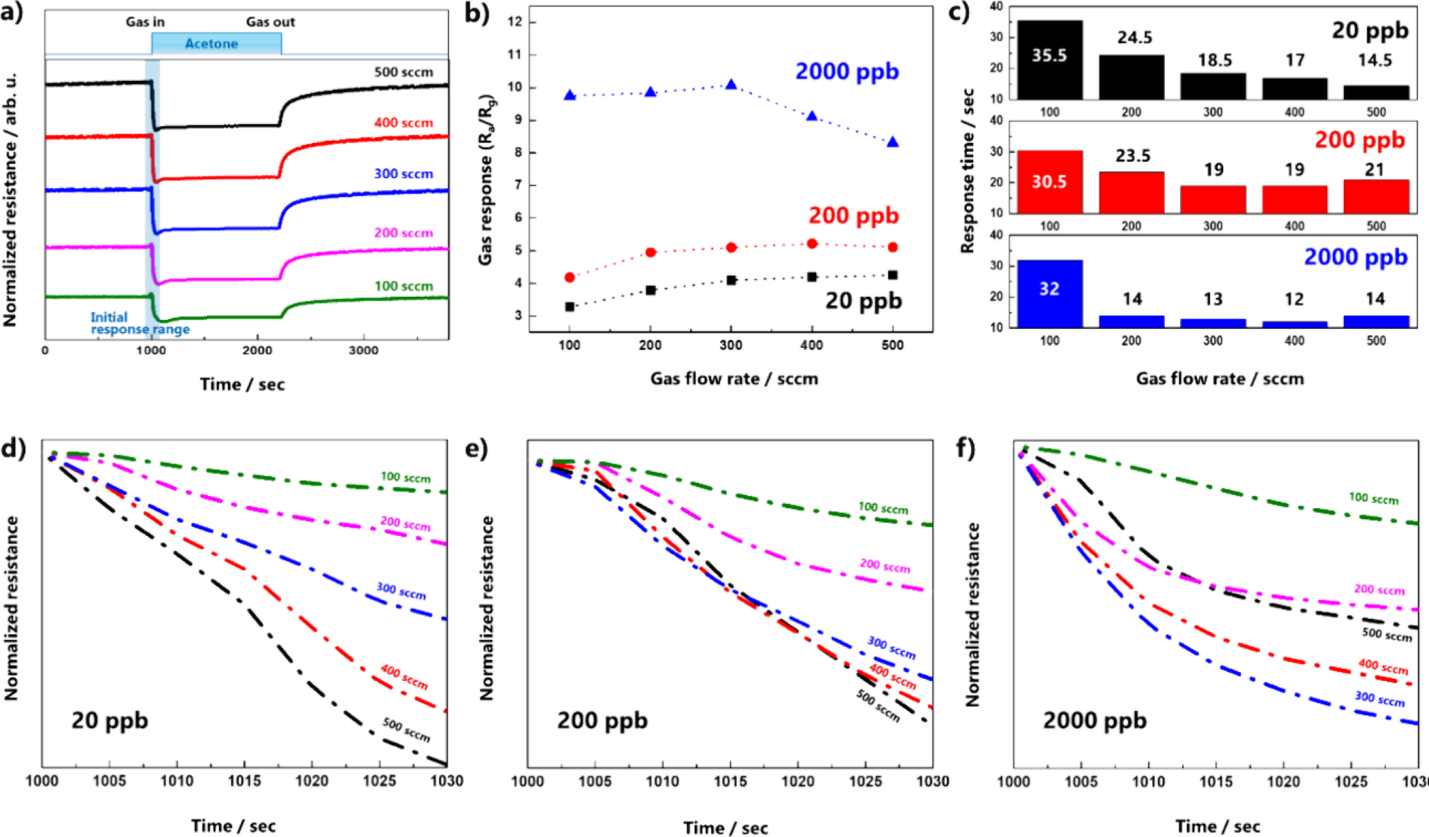
(a) Dynamic responses curves, (b) gas response values, (c) response times of the SnO_2_ nanosheet depending on the gas flow rate and concentration, and (d–f) normalized resistance variations for the initial response range (∼30 s) for 20, 200, and 2000 ppb with the gas flow rate.

The effects of the flow rate and concentration on gas detection were investigated. The dynamic curves of the SnO_2_ gas sensor were measured at various flow rates (100, 200, 300, 400, and 500 sccm) and concentrations (20, 200, and 2000 ppb). From the dynamic curve at 20 ppb (Figure 4a), variation in the gas response values with the flow rate was confirmed. The variation in the gas responses under all conditions is presented in Figure 4b. A high gas response value was observed at high concentrations of 20 and 200 ppb, whereas the effect of the flow rate was quite different in the case of 2000 ppb. At 2000 ppb, the maximum gas response value was observed at 300 sccm, after which it decreased rapidly with an increasing flow rate. Response time (τ_res_), a time required to reach 90% of the maximum response during exposure to the target gas, is demonstrated in Figure 4c.^22^ Regardless of gas concentration, the response times were greater than 30 s at a gas flow rate of 100 sccm. In other cases, the response time decreased as the flow rate increased. In particular, it exhibited a very short response time of 12–14 s at 2000 ppb. To reveal the initial molecular reaction behavior, we focused on 30 s of the initial response, as marked in Figure 4a. The resistance changes in the initial response for different concentrations are presented in Figure 4d–f. The initial response range at 20 ppb confirmed that the slope became steeper with an increasing flow rate. However, this behavior changed with increasing gas concentration. The dynamic curves at 300, 400, and 500 sccm overlapped for 200 ppb. Moreover, the trend reversed at the highest concentration; the line at 300 sccm decreased rapidly and showed the largest variance compared with those at 400 and 500 sccm. At 100 sccm, it appears to be independent of the gas concentration. This initial response variation might indicate that the gas concentration and flow rate have a complex effect on the change in resistance. As mentioned earlier, the initial change behavior is an important factor in inferring the performance because the reaction resistance is determined by the reduction of preadsorbed oxygen by acetone and the adsorption of fresh oxygen. However, the results shown in Figure 4d–f can only be qualitatively discussed.

**Figure 5:**
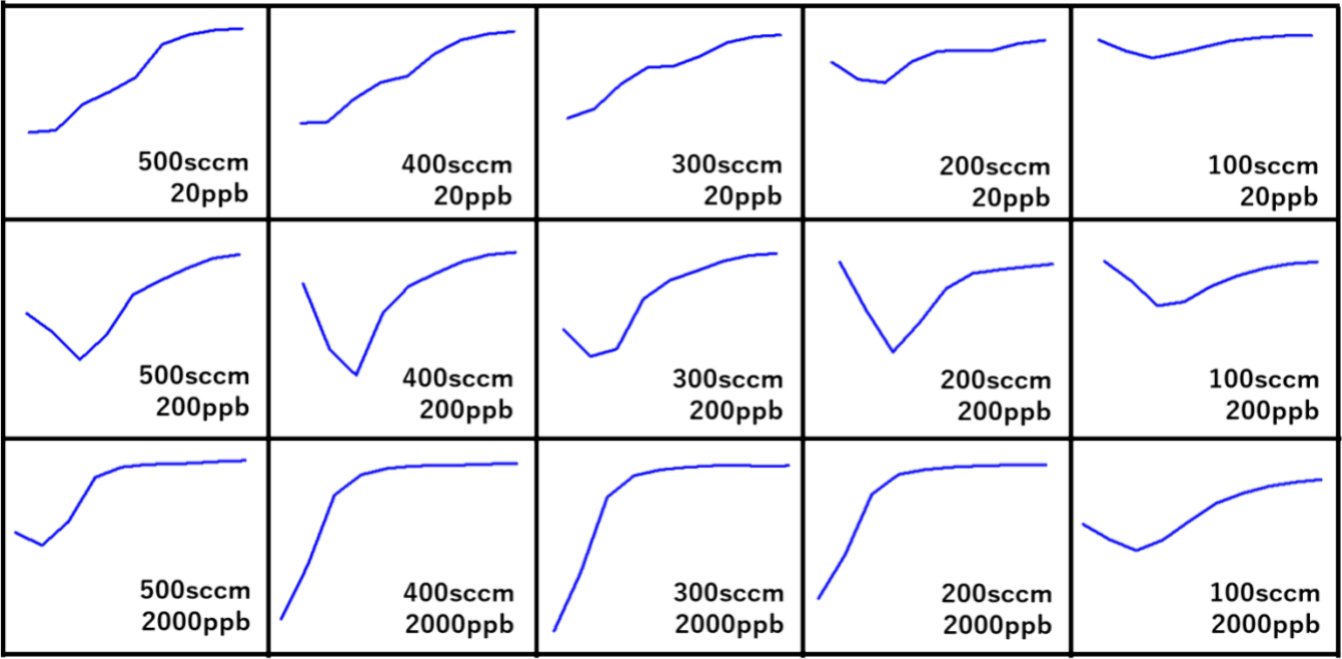
Differential graph of resistance variation in the initial response range with different gas flow rates and concentrations.

Although the trend of the response with the concentration and flow rate was approximately revealed, a more definite analysis is required to precisely understand the molecular behavior under specific conditions. Therefore, the resistance changes were converted to resistance change rates through differentiation, as shown in Figure 5, and certain patterns were observed. At a low concentration of 20 ppb, the rate of change gradually decreased and exhibited a linear variation. V-shaped lines with minimum points in the middle of the variation were observed at 200 ppb. In addition, rapidly decreasing and saturated graphs of different types were obtained at 2000 ppb.

**Figure 6:**
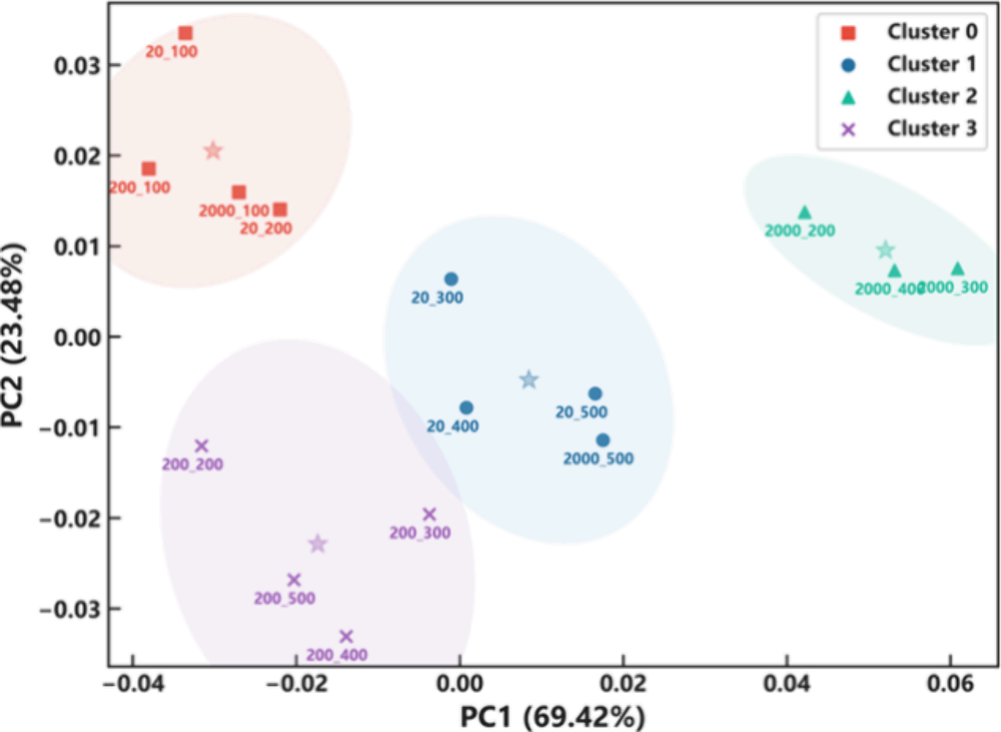
Clustered score plot after applying K-means clustering.

Although a specific pattern was confirmed based on the conditions, further statistical analyses were performed to obtain a more quantitative classification. First, PCA was used to obtain a simple visualization. This method reduces the dimensionality of the data set for a facile interpretation. This is achieved by calculating the orthogonal eigenvectors (principal components, PC) that lie in the direction of maximum variance within the data set. The highest degree of variance was provided by the first PC, and the other PCs were determined sequentially, depending on the order of variance contribution. Thus, for the most significant variances in the data in a lower-dimensional space, simple data visualization can be performed. Before PCA was performed, the data were normalized based on their concentrations. The normalized data were in the [-1,0] range. It was then subjected to PCA, and the result is shown in a score plot in Figure S1, where the eigenvalue and covariance matrix are listed in Tables S1 and S2, respectively. The resulting 2D PCA explained the variance of the data at 69.42 and 23.48%. This implies that approximately 95% of the information in the data can be represented using only two dimensions from the multidimensional original data. In this plot, some points lie near each other, indicating their similarity. Some points were completely isolated from the others. K-means clustering was introduced to group them based on similarity. Based on this algorithm, the number of predefined clusters was set from two to six to obtain different results. The quality of clustering was validated by the elbow method using two matrices: WSS and CH index. WSS is a measure of the compactness of clusters in K-means clustering. A smaller WSS indicates that the clusters are more tightly packed and, thus, more distinct from one another. The results are shown in Figure S2a. The WSS decreased as the number of clusters increased. For a total of four clusters, the rate of decrease in the WSS starts to level off, indicating that this is the optimal number of clusters. Moreover, the CH index was calculated for different numbers of clusters as an alternative measure for evaluating the quality of clustering in addition to the WSS metric. The CH index is based on the ratio of between-cluster variance to within-cluster variance and measures how well the data points are separated into distinct clusters. The elbow plot of the CH varied with the cluster number, as shown in Figure S2b. The highest value of CH at 37.08 was obtained for four clusters, indicating that this was the optimal cluster number. The validation using these two matrices yielded consistent results. Therefore, the data were divided into four groups, which then were plotted as a clustered score plot of the first two PCs with 95% confidence ellipses, as shown in Figure 6. The centroid of each cluster is represented by a star. Four groups were labeled from cluster 0 to cluster 3, with the number of cluster members being 4, 4, 3, and 4, respectively. The pattern of each cluster is shown in Figure S3, and the cluster members are listed in Table 1. Based on the data analysis, the characteristics of gas sensing were divided into four groups.

**Table 1:** Gas Response Process Type Depending on the Gas Concentration and Flow Type

**type**	**gas concentration [ppb]**	**flow rate [sccm]**	**response time [s]**	**response process**
1	20	300, 400, 500	14 < *t* < 20	moderate acetone reaction and slow saturation
2	20	200	20 < *t* < 30	compete between oxygen and acetone and then acetone reaction becomes dominant
	200	200, 300, 400, 500		
3	2000	200, 300, 400, 500	*t* < 14	rapid acetone reaction and fast saturation
4	20, 200, 2000	100	30 < *t*	very slow reaction oxygen and acetone and then acetone reaction becomes dominant with slow rate

These visualization results help determine the dominant factor and predict the molecular reaction process for each pattern type.

Type 1: A low concentration of acetone gas with a sufficient flow rate can react gradually with the preadsorbed oxygen on the surface.

Type 2: An intermediate concentration of acetone gas with a sufficient flow rate competes with oxygen in the initial step, and then, the reduction reaction by acetone becomes dominant; thus, it shows the maximum rate before finally saturating (V-shaped curve).

Type 3: A high concentration of acetone gas with a sufficient flow rate rapidly reaches the maximum reduction rate owing to the abundance of acetone molecules. Consequently, saturation occurs early.

Type 4: All acetone gas concentrations were used at a flow rate of 100 sccm. The rate of change was quite small compared to those of the other types. This effect was also completely unaffected by the concentration.

**Figure 7:**
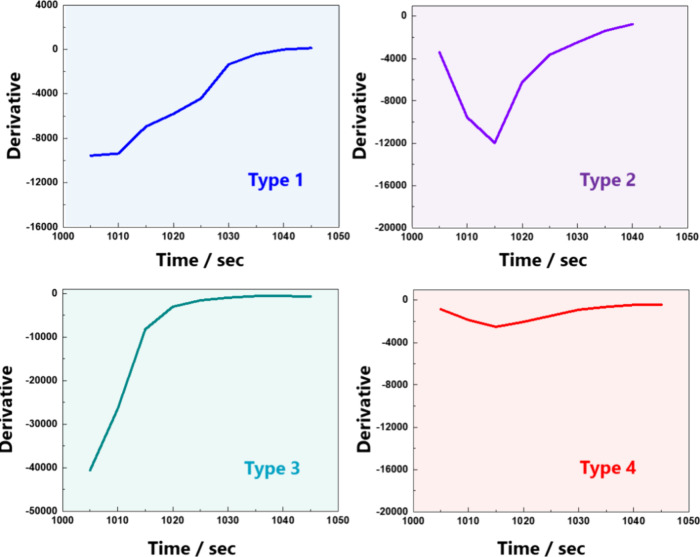
Representative pattern of differential graphs for initial response classified by K-means clustering.

Table 1 presents the expected reaction type, depending on the gas concentration and flow rate. In most cases, the reaction types were categorized by the gas concentration rather than gas flow. However, the rather slow flow rate would influence molecule reaction behavior. Especially, at 100 sccm of the gas flow rate, gas reactions were not distinguished by the gas concentration. According to this classification, it was confirmed that the response time was also categorized in the same way, as shown in Figure 4c. The fastest response time (below 14 s) and slowest response time (over 30 s) were observed in types 3 and 4, respectively. In type 1, the gas response time was between 14 and 20 s, and type 2 showed a slightly slow response time in the range of 20–30 s (Figure 7).

## Conclusions

In this study, we suggest four types of sensing categorizations depending on the gas concentration and flow rate using PCA and the K-means clustering method in an ultrahigh-sensitivity gas sensor. The as-prepared SnO_2_ nanosheet gas sensor was tested under 15 conditions in the concentration and flow rate ranges of 20–2000 ppb and 100–500 sccm, respectively. A database was created by using differential values of the gas response results for the initial 30 s. In the PCA results, two principal components were 92.9% of the total variance in data, which was sufficient to represent the data set in two dimensions. Four clusters were determined from the dimensionally compressed results based on the clustering validation. The clustering results were analyzed based on the reaction behavior between the acetone and oxygen molecules. This was categorized according to the timing at which the reaction of the acetone molecules became dominant or saturated. The categorization was mostly determined by the gas concentration rather than the flow rate, except at a flow rate of 100 sccm. In addition, the gas response time can be classified by using the suggested categorization. Our results indicate that data analysis can support the prediction of the molecular reaction between the target and oxygen molecules, which is an important mechanism of gas sensing. This involves the utilization of data analysis beyond the common optimization of experimental conditions, and this approach should be performed in the future. Our concept is sophisticated by machine-learning algorithms and deep learning using artificial neural networks and can be applied to practical user fields.

## Supporting Information Available

PCA score plot, its eigenvalue and covariance matrix from the database for the gas concentration and flow rate, and elbow plot of WSS and CH index varied with cluster numbers and cluster mean plot for clustering (PDF)

## ABBREVIATIONS

VUV: vacuum ultraviolet
FE-SEM: field-emission scanning electron microscopy
TEM: transmission electron microscopy
HR-TEM: high-resolution transmission electron microscopy
XRD: X-ray diffraction
XPS: X-ray photoelectron spectroscopy
ANOVA: analysis of variance
PCA: principal component analysis
PC: principal components
WSS: within-cluster sum of squared errors
CH: Calinski–Harabasz index
BSS: between-cluster sum of squares
