# Supplementary material for: Insights into Target Gas–Oxygen Interactions in Highly Sensitive Gas Sensors Using Data-Driven Methods
Source: ACS Sens. 2024 Nov 12;9(11):5848–55. doi: 10.1021/acssensors.4c01284 (PMC11590098; doi:10.1021/acssensors.4c01284)
Supplement: Supplementary file 1 — se4c01284_si_001.pdf [file se4c01284_si_001.pdf]

Supporting information

Insights into target gas-oxygen interactions in highly sensitive gas sensors using data-driven methods

Kyusung Kim[1], Phuwadej Pornaroontham[2], Hojung Yun[3], Sungmin Kim[4], Pilgyu Choi[5], Yoshitake Masuda[5*]

[1] Institute of Material Innovation, Institutes of Innovation for Future Society, Nagoya University, Nagoya 464-8601, Japan

[2] Department of Chemical Technology, Faculty of Science, Chulalongkorn University, Bangkok 10330, Thailand

[3]Department of Sustainable Materials and Technology for Industries, Faculty of Engineering, Nagoya University, Nagoya 464-8601, Japan

[4]Korea Institute of Industrial Technology, Surface R&D group, 156, Gaetbeol-ro, Yeonsu-gu, Incheon 21999, Republic of Korea

[5] National Institute of Advanced Industrial Science and Technology (AIST), 2266-98 Anagahora, Shimoshidami, Moriyama, Nagoya 463-8560, Japan

KEYWORDS: Nanosheets, Gas sensors, K-mean clustering, Principal component analysis

Corresponding author: masuda-y@aist.go.jp (Yoshitake Masuda)

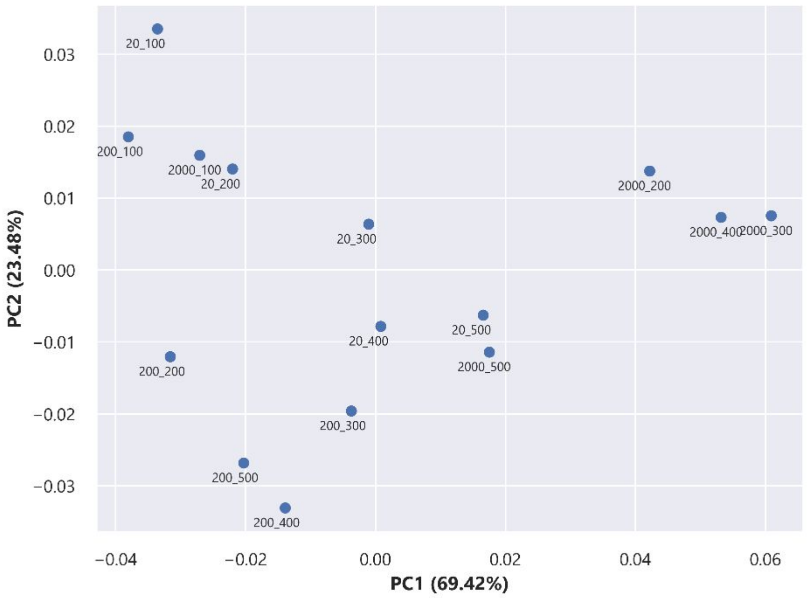

Fig.S1 PCA scoreplot

Number	Eigenvalue	%	Sum %
1	1.02E-03	69.42	69.42
2	3.45E-04	23.48	92.90
3	5.80E-05	3.95	96.84
4	1.63E-05	1.11	97.95
5	1.23E-05	0.84	98.79
6	5.04E-06	0.34	99.14
7	4.47E-06	0.30	99.44
8	3.00E-06	0.20	99.64
9	2.31E-06	0.16	99.80
10	1.15E-06	0.08	99.88
11	9.97E-07	0.07	99.95
12	5.86E-07	0.04	99.99
13	1.89E-07	0.01	100.00
14	1.54E-08	0.00	100.00
15	1.74E-35	0.00	100.00
	1.47E-03	100.00	

Table S1. Eigenvalue

	950	955	960	965	970	975	980	985	990	995	1000	1005	1010	1015	1020	1025	1030
950	1.05E-05	2.50E-06	-3.58E-06	3.64E-06	1.30E-06	-2.54E-06	1.96E-06	-3.49E-06	-3.55E-06	3.09E-06	1.75E-05	1.21E-05	-1.10E-05	-7.72E-06	-3.74E-07	-1.87E-06	-3.66E-06
955	2.50E-06	2.26E-06	-1.78E-07	-1.64E-07	3.98E-07	2.59E-07	7.93E-07	-6.89E-07	-1.37E-06	-1.16E-06	-3.30E-06	-5.54E-07	2.69E-06	2.65E-06	4.29E-06	2.20E-06	4.63E-07
960	-3.58E-06	-1.78E-07	2.40E-06	-1.18E-06	-3.56E-07	1.43E-06	-4.92E-07	7.57E-07	1.59E-06	-1.41E-06	-7.03E-06	-4.44E-07	1.01E-05	6.57E-06	1.47E-06	1.15E-06	1.50E-06
965	3.64E-06	-1.64E-07	-1.18E-06	3.15E-06	9.43E-07	-1.46E-06	3.23E-07	-2.15E-06	-1.21E-06	2.84E-06	1.87E-05	1.46E-05	-5.57E-06	-3.64E-06	-2.26E-06	-3.03E-06	-2.86E-06
970	1.30E-06	3.98E-07	-3.56E-07	9.43E-07	1.82E-06	8.00E-07	-6.31E-07	-1.69E-06	-8.32E-07	8.45E-07	1.12E-05	7.73E-06	-3.96E-06	4.25E-07	1.83E-06	5.89E-08	-4.19E-07
975	-2.54E-06	2.59E-07	1.43E-06	-1.46E-06	8.00E-07	3.15E-06	7.65E-08	6.74E-07	1.76E-06	-2.63E-06	-6.63E-06	4.10E-06	1.54E-05	1.12E-05	6.54E-06	4.58E-06	3.52E-06
980	1.96E-06	7.93E-07	-4.92E-07	3.23E-07	-6.31E-07	7.65E-08	3.14E-06	4.18E-07	-1.12E-06	-9.93E-08	7.90E-06	1.70E-05	9.30E-06	6.42E-07	5.62E-07	-6.57E-08	-1.18E-06
985	-3.49E-06	-6.89E-07	7.57E-07	-2.15E-06	-1.69E-06	6.74E-07	4.18E-07	3.58E-06	2.98E-06	-3.29E-06	-1.22E-05	-1.49E-06	1.20E-05	3.61E-06	6.43E-07	1.84E-06	1.83E-06
990	-3.55E-06	-1.37E-06	1.59E-06	-1.21E-06	-8.32E-07	1.76E-06	-1.12E-06	2.98E-06	7.36E-06	-3.33E-06	-2.74E-05	-1.11E-05	2.15E-05	1.50E-05	6.04E-06	4.77E-06	4.15E-06
995	3.09E-06	-1.16E-06	-1.41E-06	2.84E-06	8.45E-07	-2.63E-06	-9.93E-08	-3.29E-06	-3.33E-06	7.25E-06	2.81E-05	9.07E-06	-2.42E-05	-1.41E-05	-7.88E-06	-7.59E-06	-6.26E-06
1000	1.75E-05	-3.30E-06	-7.03E-06	1.87E-05	1.12E-05	-6.63E-06	7.90E-06	-1.22E-05	-2.74E-05	2.81E-05	4.97E-04	4.50E-04	-1.00E-04	-1.29E-04	-7.88E-05	-6.17E-05	-5.46E-05
1005	1.21E-05	-5.54E-07	-4.44E-07	1.46E-05	7.73E-06	4.10E-06	1.70E-05	-1.49E-06	-1.11E-05	9.07E-06	4.50E-04	5.36E-04	5.55E-05	-7.07E-05	-5.53E-05	-4.36E-05	-4.24E-05
1010	-1.10E-05	2.69E-06	1.01E-05	-5.57E-06	-3.96E-06	1.54E-05	9.30E-06	1.20E-05	2.15E-05	-2.42E-05	-1.00E-04	5.55E-05	2.06E-04	1.09E-04	5.01E-05	3.33E-05	2.27E-05
1015	-7.72E-06	2.65E-06	6.57E-06	-3.64E-06	4.25E-07	1.12E-05	6.42E-07	3.61E-06	1.50E-05	-1.41E-05	-1.29E-04	-7.07E-05	1.09E-04	1.09E-04	6.39E-05	3.64E-05	2.36E-05
1020	-3.74E-07	4.29E-06	1.47E-06	-2.26E-06	1.83E-06	6.54E-06	5.62E-07	6.43E-07	6.04E-06	-7.88E-06	-7.88E-05	-5.53E-05	5.01E-05	6.39E-05	4.81E-05	2.65E-05	1.47E-05
1025	-1.87E-06	2.20E-06	1.15E-06	-3.03E-06	5.89E-08	4.58E-06	-6.57E-08	1.84E-06	4.77E-06	-7.59E-06	-6.17E-05	-4.36E-05	3.33E-05	3.64E-05	2.65E-05	1.87E-05	1.30E-05
1030	-3.66E-06	4.63E-07	1.50E-06	-2.86E-06	-4.19E-07	3.52E-06	-1.18E-06	1.83E-06	4.15E-06	-6.26E-06	-5.46E-05	-4.24E-05	2.27E-05	2.36E-05	1.47E-05	1.30E-05	1.15E-05

Table S2. Covariance matrix

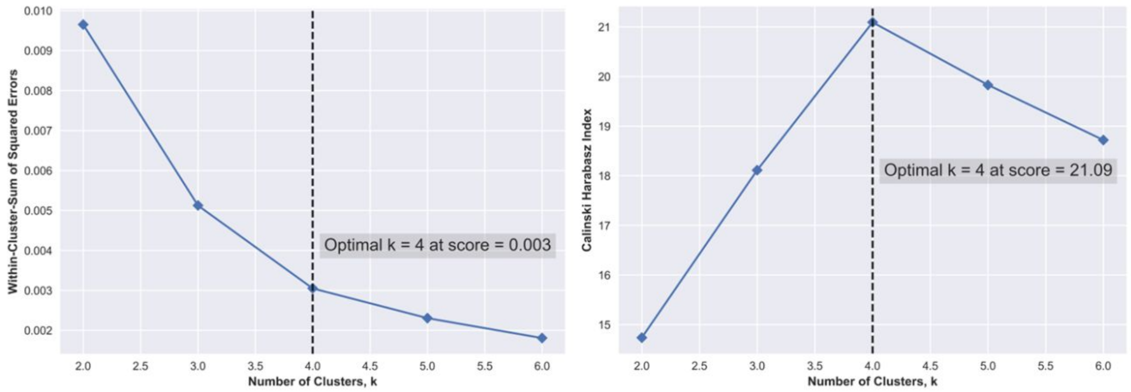

Fig.S2 Elbow plot of a) WSS and b) CH index varied with cluster numbers

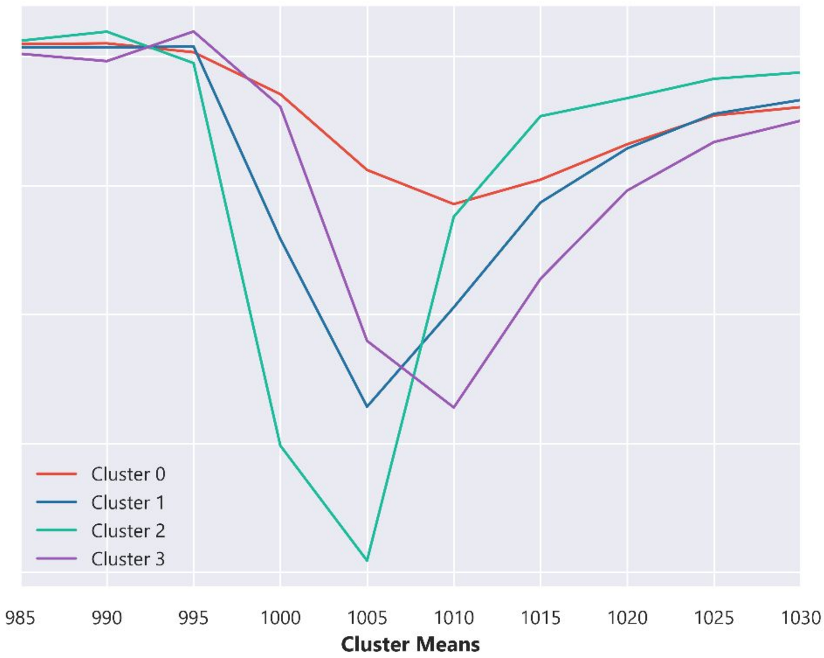

Fig.S3 Cluster Means plot.
